# Septicémie fatale due à une bactérie émergente: arcanobactrium hemolyticum

**DOI:** 10.11604/pamj.2016.25.28.6948

**Published:** 2016-09-27

**Authors:** Mohammed Frikh, Abdelhay Lemouer, Mustapha Moutawakil, Adil Maleb, Mostafa Elouennass

**Affiliations:** 1 Laboratoire de Bactériologie, Université Mohammed V, Faculté de Médecine et de Pharmacie, Hôpital Militaire d’Instruction Mohammed V, Rabat, Maroc; 2 Faculté de Médecine et de Pharmacie, Mohammed I Université d’Oujda, Maroc

**Keywords:** Arcanobacterium hoemolyticum, immunodépression, septicémie, sensibilité, Arcanobacterium haemolyticum, immunosuppression, sepsis, sensitivity

## Abstract

L’*Arcanobactérium haemolyticum (A.haemolyticum)* est un bacille à Gram positif. L’homme en est le principal réservoir. C’est un pathogène opportuniste essentiellement chez l’immunodéprimé, et qui peut être responsable d’infection au niveau de la peau et du pharynx chez les sujets sains, surtout chez les enfants et les adolescents. Il peut causer des surinfections des ulcères chroniques, et occasionnellement des infections invasives. Sa détection au niveau des cultures reste toujours difficile car il simule de nombreuses bactéries auxquelles il est souvent associé dans les produits pathologiques. Et Il n’existe pas de recommandations concernant l’étude sa sensibilité aux antibiotiques. Les bactériémies à *Arcanobactérium* sont rares, à notre connaissance, seize cas ont été décrits dans la littérature. Nous rapportons, un autre cas de bactériémie à *A.haemolyticum*, secondaire à une surinfection d’escarres fessières.

## Abstract (en)

Arcanobacterium hemolyticum (A.haemolyticum) is a gram-positive bacillus. Man is the primary environmental reservoir. It is essentially an opportunistic pathogen in immunocompromised patients and may be responsible for infections of the skin and pharynx in healthy subjects, especially in children and adolescents. It can cause superinfections of chronic ulcers, but occasionally it causes invasive infections. Its isolation from culture samples is always difficult because it simulates many bacteria to which it is often associated in pathological products. There are not recommendations concerning the study of its antibiotics sensitivity. Arcanobacterium Bacteremia are rare to our knowledge, only sixteen case reports have been described in the literature. We here report another case of a patient with A.haemolyticum bacteremia secondary to superinfection of gluteal eschars.

## Introduction

L’*Arcanobactérium haemolyticum (A.haemolyticum)* est un bacille à Gram positif. L’homme en est le principal réservoir, cependant, on peut le rencontrer aussi chez l’animal [1]. C’est un pathogène opportuniste chez l’immunodéprimé en particulier chez les séropositifs ou VIH [2], par ailleurs il peut être responsable d’infection chez l’immunocompétent [3]. Il a été isolé au niveau de la peau et du pharynx chez les sujets sains, chez lesquels il est responsable de 0,5 à 2,5% des pharyngites principalement chez les enfants et les adolescents [2]. Il peut causer des surinfections des ulcères chroniques chez les diabétiques, et occasionnellement des infections invasives [4]. Le diagnostic des infections à *Arcanobactérium* reste difficile, car les colonies qu’il produit, simulent celles des streptocoques ou des *corynébactéries* [2], d’autant plus que leur association est fréquente. Les bactériémies à *Arcanobactérium* sont rares, à notre connaissance, seize cas ont été décrits dans la littérature. Nous rapportons, un autre cas de Bactériémie à *A.haemolyticum*, secondaire à une surinfection d’escarres fessières.

## Patient et observation

Mr H. KH âgé de 70 ans, admis dans le service de chirurgie réparatrice, pour prise en charge d’escarres fessières et trochantériennes, secondaire à une paraplégie post traumatique. Deux jours après, il a été transféré au service de réanimation suite à une détérioration de son état général. A l’admission, le patient était comateux avec un Glasgow coma score à 9/15, paraplégique, fébrile à 39°C, dyspnéique, présentant un ictère cutanéo-muqueux, une ascite, un œdème des membres inferieurs, et d’énormes escarres sacro fessières et trochantériennes.

Une série de 3 hémocultures a été effectuée, puis incubée dans l’automate BACTEC 9240 (Beckton Dickinson Microbiology Systems Ltd, USA). 36 heures après elles se sont positivées. La subculture sur milieux gélosés au sang de cheval à 5% et incubés à 37°C et en atmosphère riche en CO2, ont permis l’isolement de fines colonies béta-hémolytiques. L’examen direct après coloration de Gram, a montré des bacilles à Gram positif pléomorphes, la catalase était négative. Une galerie API coryné^®^ (Bio- Mérieux, Marcy l’étoile France) a permis l’identification d’*Arcanobacterium haemolyticum* à 99.9% avec une excellente identification (code 6530361). De même, L’examen cytobactériologique des échantillons obtenus à partir des escarres, a permis l’isolement et l’identification de la même bactérie dans les mêmes conditions ci dessus.

L’étude de la sensibilité des deux isolats, par méthode de diffusion des disques selon les recommandations de l’EUCAST, a montré le même profil de sensibilité : Sensible à l’oxacilline 5μg, amoxicilline, gentamicine 500 μg, érythromycine, lincomycine, Tétracycline, Rifampicine, teicoplanine, vancomycine; résistant à la lévofloxacine et la moxifloxacine, et intermédiaire à la Pristinamycine. L’étude de la CMI de l’amoxicilline par E-test était de 0.15mg /l.

Le bilan biologique a montré une hypo protidémie importante à 20 g/l et une hypo albuminémie sévère à 15g/l, l’électrophorèse des protides a révélée un effondrement des gammaglobulines IgG à 5g/l. La CRP était à 291,3 mg/l. L’hémogramme a montré, des leucocytes à 5300/μl, une hémoglobine à 7g/dl. Le temps de Quick TP à 51%, le TCA 48,9sec pour un témoin de 32sec, le fibrinogène à 5,71g/l.

Le patient à été mis sous amoxicilline-acide clavulanique 1g toute les 8 heures par voie intra veineuse. Néanmoins, l’évolution était fatale 5 jours après, dans un tableau de sepsis grave malgré la sensibilité de la bactérie à l’antibiotique utilisé.

## Discussion

*A.haemolyticum* est un bacille à gram positif parfois à Gram variable pléomorphique avec ou sans ramifications. Cette bactérie a été classée initialement dans le genre *Corynébactérium*, lors de sa découverte en 1946 [5]. En 1982, elle a été reclassée dans un nouveau genre *Acranobactérium* qui comprend six espèces: *A.haemolyticum, A.pyogenes, A.bernardiae, A.phocae, A.pluranimalium, et A.hippocoleae* [1]. Ce microorganisme est souvent considéré comme une bactérie commensale, faisant partie de la flore normale obligatoire du pharynx, des voies aériennes supérieures et de la peau [6]. Il n’existe aucun facteur de risques d’infection pour cette bactérie. Cependant elle peut être responsable d’infection du tractus respiratoire supérieur chez les jeunes en bonne santé, et des infections de la peau et des tissus mous chez des patients plus âgées et souvent immuno-compromis [1].

Depuis sa description, *A. haemolyticum* a été incriminé dans de nombreuses infections telles que : les infections de la peau (les cellulites), ostéomyélite, syndrome de Lemière, abcès thyroïdiens, pharyngite aigue et exanthème en particulier chez les enfants et les jeunes adultes [1, 6], La plus forte incidence s’observe chez les individus entre 15 et 18 ans [7]. Les cas de bactériémies sont rares, elles surviennent aussi bien chez les immunocompétents, que chez les immuno- compromis, c’est le cas de notre patient, qui avait une hépatopathie sévère, avec un taux de protides à 20g/l, associé à une hypogammaglobulinémie sévère. L’immunité humorale défaillante chez notre patient pourrait être un des facteurs favorisants cette infection invasive, auquel s’ajoute la virulence potentielle de cette espèce [8].

En effet l’étude génotypique montre que *A haemolyticum* possède, sept gènes de virulence potentielle, qui codent pour diverses enzymes: acrolysine, phospholipase D, hémolysine A, neuraminidase A et neuraminidase, H protéines de la famille facteurs CAMP, protéine de liaison au collagène ; ces gènes sont inconstamment présents au niveau du chromosome bactérien [8] ce qui explique le polymorphisme des manifestations cliniques. Les bactériémies à *A. haemolyticum* peuvent avoir des points de départ divers: lésion pulmonaire, infection cutanée et des tissus mous, pharyngites, amygdalites aigues, ulcères de la peau [1, 6]. Pour notre patient, la porte d’entrée était les escarres fessières. En effet la même souche à été isolée aussi bien dans les hémocultures que dans les prélèvements des tissus nécrosés. La reconnaissance de cette bactérie dans les cultures, peut être délicate pour plusieurs raisons: d’une part la croissance peut être supplantée par les autres bactéries à pousse rapide, d’autres part, l’aspect macroscopique en culture, sur gélose au sang, prête confusion avec d’autres microorganismes tels que, les streptocoques béta hémolytiques et les *corynébatéries* [1, 5]. Le caractère hémolytique peut être mis en évidence sur des géloses enrichies de sang de mouton, ou du cheval mais il semble que celui-ci donne un meilleur rendement [5]. Il peut être optimisé par l’incubation en atmosphère enrichie de 10% de CO2 car les hémolysines secrétées par les bactéries sont oxygéno-labiles.

Après culture, deux types de colonies peuvent être observées: des colonies lisses qui prédominent dans les infections pyogènes ; c’est le cas des isolats chez notre patient et les colonies rugueuses qui sont presque exclusivement isolées à partir des infections du tractus respiratoire [3]. L’agglutination avec l’antisérum de *streptocoque* B est une autre cause d’erreur en matière d’identification de cette bactérie [1]. L’identification par les galeries API *coryné* ne présente pas de difficulté particulière. Dans une étude comparative entre ces galeries et la spectrométrie de masse [9], il s’est avéré qu’il existe une excellente corrélation. Toutes les souches identifiées par API *Coryne* V2.0 l’étaient aussi par MALDI-TOF MS [9]. L’identification de notre souche était excellente par galerie API *coryné* à 99,9%. Le CAMPS test (synergie entre l’hémolysine *A. haemolyticum* et *Rhodococcus equi* ou groupe streptocoque B) peut être aussi utilisé comme complément pour l’identification, mais qui n’a pas été réalisé dans notre cas.

Concernant l’étude de la sensibilité de l’*A. haemolyticum*, il n’existe pas de recommandations établies. Les souches testées dans la littérature, étaient sensibles aux pénicillines, cephalexine, erythromycine, clindamycine et gentamicine; mais résistant aux sulfamethoxazole-trimethoprime [10]. Toutefois, Les céphalosporines constituent le traitement de choix. Les résultats obtenus en utilisant n’importe quelle méthode (méthode de diffusion, E test) doivent être interprétés avec prudence [10]. Même si notre souche était sensible à la molécule utilisée dans le traitement, l’issue fatale de notre patient pourrait être due au terrain débilité et probablement à la virulence potentielle de cette souche.

## Conclusion

Les infections à *A. haemolyticum* et en particulier les bactériémies sont rares. Leur diagnostic est parfois difficile. La difficulté de leur mise en évidence pose un réel problème et retarde parfois la prise en charge. Par ailleurs, d’autres études sont nécessaires pour standardiser son antibiogramme.
